# Emotional Intelligence (EI) Training Adapted to the International Preparation Constraints in Rugby: Influence of EI Trainer Status on EI Training Effectiveness

**DOI:** 10.3389/fpsyg.2019.01939

**Published:** 2019-09-20

**Authors:** Mickaël Campo, Sylvain Laborde, Guillaume Martinent, Benoît Louvet, Michel Nicolas

**Affiliations:** ^1^Laboratory of Psychology, Psy-DREPI (EA7458), Faculty of Sport Sciences, Université Bourgogne Franche-Comté (UBFC), Dijon, France; ^2^Department d’Accompagnement Mental à la Performance, Fédération Française de Rugby (FFR), Marcoussis, France; ^3^Department of Performance Psychology, German Sport University Cologne, Cologne, Germany; ^4^CESAMS (EA4260), UFR STAPS, Normandie Université, Caen, France; ^5^L-VIS (EA 7428), Faculty of Sports Sciences, Université Claude Bernard Lyon 1, Lyon, France; ^6^CETAPS (EA 3832), Faculty of Sports Sciences, Normandie Université, Rouen, France

**Keywords:** emotional intelligence, training, coaching, team sports, rugby, performance, elite sport, coach-athlete relationship

## Abstract

Given the positive influence of emotional intelligence (EI) on sports performance, particular attention should be paid on how to improve it. Following promising results, previous research concluding that it was possible to improve EI *via* specific training programs also raised considerable debates. Indeed, previous EI training programs were very time-consuming for participants. This lessens consequently their suitability with the schedule constraints of elite sport. While, in the absence of sport psychologists, numerous coaches or physiologists try to work with players to improve their emotional competences, the aim of this study was to investigate the effectiveness of EI training programs fitting the schedule constraints of elite team sports, provided by three different EI trainers: the team’s coach, the team’s physiotherapist, and an expert in sport psychology. Young elite rugby union players (*N* = 96) participated in this study. Based on schedule constraints imposed by the head coach of the French u18 rugby union national team, the program consisted in three 1 h group-based EI training sessions occurring the last 3 days before a game (1 per day). Linear mixed-effects models showed that despite the constraining organizational challenge imposed by the coach, the intervention helped the players to increase some emotional competences at the trait level. Furthermore, a pairwise analysis showed that the type of emotional competencies developed depended on the status of the EI trainers. These findings highlight the suitability of a group-based approach in the training-week structure. They also point the way to EI improvement in a short period of time. Moreover, the specific influences of the EI trainer’s status on players’ EI development invite coaches and researchers to jointly combine their efforts in order to increase the EI training opportunities and to maximize the effects of their interventions. Together, these preliminary results provide first evidence facilitating the integration of such work in the preparation periods during international seasons.

## Introduction

Emotions play a key role in sport performance (e.g., Lane et al., 2010; Laborde et al., 2013; Doron and Martinent, 2017; Martinent and Nicolas, 2017; Campo et al., 2018; Martinent et al., 2018), and rugby is no exception (Campo et al., 2012). Indeed, recent studies showed the influence of individual (Campo et al., 2016a), group-based (i.e., emotion as member of a group) and team-referent emotions (i.e., emotions of the group as a whole; Campo et al., 2019), as well as the importance of interpersonal emotion regulation processes (Campo et al., 2017). Therefore, the influence of emotional intelligence (EI) on rugby performance may occur *via* its effects at the individual and team levels (Meyer and Zizzi, 2007; Laborde et al., 2016; Kopp and Jekauc, 2018).

Indeed, although the influence of emotions have often been considered as states, it is also acknowledged that athletes should develop stable emotional competences, such as the ability to regulate ones’ own emotions (Lazarus, 2000). This trait perspective has received increased attention with the concept of EI, that is, how individual deals with their own and others’ emotions through five main emotional competences: identification, expression, understanding, regulation, and use (Petrides, 2009; Brasseur et al., 2013). Interestingly, previous research has shown that it is possible to train EI, as indicated by a recent meta-analysis (Hodzic et al., 2017) and a systematic review (Kotsou et al., 2019). EI training programs have already been tested in sports and in rugby in particular (Campo et al., 2016b). However, some limitations appeared in implementing such programs in regards with the organizational constraints of elite sports in terms of duration and feasibility. The current study aimed at addressing this issue by investigating the tripartite model (Mikolajczak, 2009), which considers three levels: the knowledge level (i.e., what people know about emotions), the ability level (i.e., what people can do regarding emotions), and the trait level (i.e., what people actually do on an everyday basis regarding emotions). Basically, EI training suggests that acting on the knowledge and ability levels would provoke changes at the trait level (Campo et al., 2015, 2017; Hodzic et al., 2017; Laborde et al., 2017a, 2018a; Kotsou et al., 2019).

EI training has already been implemented in sports teams, like in cricket (Crombie et al., 2011) and in netball (Barlow and Banks, 2014). In rugby, an EI training program has been specifically elaborated by Campo et al. (2017) comprising four individual intervention sessions lasting from 45 to 90 min, one session every 5 weeks, over one season. This EI training program was based on trait emotional intelligence theory (Petrides, 2009), and integrated aspects linked to appraisal theories (Lazarus, 1999) and the individual zone of optimal functioning in sport (Hanin, 2000). Together, those EI training programs were able to increase EI to some extent (Crombie et al., 2011; Campo et al., 2016b) or influence aspects related to emotions, such as self-efficacy and anxiety (Barlow and Banks, 2014). However, EI training programs could be usually seen as very time-consuming and not adapted to the schedule constraints of elite athletes. The current study aimed to address this gap.

Furthermore, given the frequent absence of permanent sport psychologists within the staff of elite rugby teams, mental work with players is frequently provided by the coach or the physiologist. Thus, beyond the schedule constraints, working with elite athletes in team sports raises the question of the members of a staff who deliver such intervention. For instance, we can suggest that whether the person has or has not an influence on the selection process (e.g., coach vs. physiotherapist) or whether the person is considered by the players as being an expert in sport psychology (e.g., the sport psychologist vs. other staff members) may influence the effectiveness of the EI training program. Indeed, participants’ engagement and commitment into an EI program may be contingent on their interaction with the person providing the intervention, in terms of personality traits (Cuperman and Ickes, 2009), but also in terms of the relationship with the person, which may be influenced by the status. But surprisingly, the influence of the status of the person delivering the intervention on the intervention effectiveness has been so far under-researched (Allen and Laborde, 2014). In a recent meta-analysis, Brown and Fletcher (2017) indicated that the type of provider is a perplexing issue when considering the effects of psychological interventions on sport performance. Among the very few studies addressing this topic, the type of provider accounted for some of the variation in the observed effect. Interestingly, the effects of coach-delivered interventions were higher than those provided by qualified practitioners. Given this issue still needs to be investigated, the second aim of this study was thus to clarify this influence.

To sum up, the aim of this study was to investigate the effectiveness of an EI training program fitting the schedule constraints of international rugby players and included within the training week preceding a competition like usual technical or physical conditioning sessions. The second aim of this study was to investigate whether the status of the person delivering the intervention influenced its effectiveness. First, it was hypothesized that regardless of the person delivering the intervention, the current EI training increased players’ emotional competences (based on Campo et al., 2017). In the second hypothesis, it was postulated that, compared with a rugby coach or a physiologist, the current EI training is more effective when the person delivering the intervention is an expert in sport psychology.

## Materials and Methods

### Participants

Ninety-six male 17 years old elite rugby union players were involved in this study. The players were randomly assigned within four groups: a control group (GC, *n* = 23); a first experimental group (G1, *n* = 23) in which the intervention was delivered by the first author which is an expert in sport psychology and in rugby; a second experimental group (G2, *n* = 24) in which the intervention was delivered by a the head coach of the u18 French team (rugby coach for 36 years at the young international levels and at the professional level); and a third experimental group (G3, *n* = 22) in which the intervention was delivered by a member of the medical staff of the French team (physiologist for 10 years, working with rugby teams at the professional and young international levels).

#### Measure

In this study, we used the Profile of Emotional Competences (PEC; Brasseur et al., 2013), which contains 50 items assessing five core emotional competencies separately, distinctly for one’s own and others’ emotions. It thus provides 10 subscores (identification of one’s emotions, identification of others’ emotions, understanding of one’s emotions, understanding of others’ emotions, expression of one’s emotions, listening to others’ emotions, regulation of one’s emotions, regulation of others’ emotions, use of one’s emotions, and use of others’ emotions). In line with the trait conceptualization of emotional intelligence (Petrides, 2009), this tool clearly posits the fact that emotional intelligence and/or competence can be trained and developed (Brasseur et al., 2013). Athletes were asked to indicate the extent to which they agreed with statements regarding their intrapersonal and interpersonal emotional competences on 5-point Likert scale ranging from strongly disagree to strongly agree. Reliability analysis performed on the four samples indicated very good internal consistency of the subscales (>0.86) as well as for the total score (>0.86). To the best of our knowledge, this study is one of the first studies using this instrument in the sport context. Previous studies relying on the PEC have been conducted in the general population (Mikolajczak and Van Bellegem, 2017).

### Procedure

The challenge of this present work was to build an EI training program adapted to the constraints imposed by the players’ availability during a usual 1-week preparation period preceding international games. Although approval from an ethics committee was not required for this non-invasive study as per applicable institutional guidelines and regulations, we sought to obtain an approval by a consortium of independent researchers in the humanities and in sport sciences verifying that the study procedures followed international ethical guidelines for research (World Medical Association Declaration of Helsinki, 2013).

**Figure 1:**
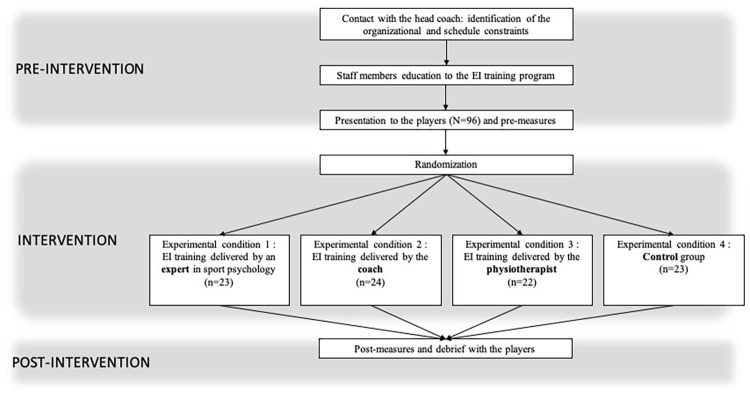
Flow chart of the experimental protocol.

As shown in Figure 1, the first stage consisted in contacting the head coach of the u18 French national rugby union team to ask him to freely schedule EI training sessions during the week preceding a game organized to select players for the two u18 French Rugby union teams. The head coach proposed to the authors to build a training program based on three 1-h sessions conducted during the three consecutive last days before the competition (1 session per day). The second stage consisted in a 3-h session to train the head coach and the team’s physiologist so as they were able to deliver the training program with players. Finally, at the beginning of the training camp, we met with the participants to provide them with information about the study. Players who agreed to participate in the study provided written informed consent.

The EI training was developed on the basis of the work by Campo et al. (2015, 2016b), adapted to the imposed organizational constraints. The two first sessions focused on the intrapersonal emotional competencies, while the last session aimed at improving interpersonal ones. Particularly, the program was conducted with examples, illustrations, videos, and exercises taken from the rugby context to facilitate the appropriation of the contents relating to emotional competences. Each session was implemented with half of the team and then again with the other half according to the rugby training schedule. That is, when the back players participated in the EI session, the forward players participated in a rugby training session, and vice versa. At the same time, the control group participated in three 1-h sessions during which the players followed video analyses of u18 competitions with a game analyst without any feedback related to emotional competences.

At the end of the three EI training sessions, the participants completed the same measure of emotional competences.

### Data Analysis

To assess the influence of the intervention and that of the person delivering the intervention, every 10 subscales and the global score of emotional competences were successively modeled by the same linear mixed-effects model (LMEM, Pinheiro and Bates, 2000) process. Particularly, two piecewise LMEMs were used to model changes in emotional competences. First, we compared the control group with experimental groups in the same models. Subjects were a random effect. Condition (control vs. experimental), linear time, and their interactions were considered as fixed effects. Second, in order to carefully detail the effect of the person providing the intervention, the analysis strategy consisted in pairwise comparisons considering each every experimental group to the control group. Subjects were a random effect. Condition (control vs. expert, control vs. coach, and control vs. physiologist), linear time, and their interactions were considered as fixed effects.

A descending selection algorithm was employed. Removal was based on likelihood ratio tests at the 5% threshold, respecting marginality restrictions (Venables and Ripley, 2002, p. 172). The final resulting model was interpreted using coefficients for each independent variable in the model, corresponding t statistics, degrees of freedom, *p* using the alpha level, and relative effect sizes (ES). All calculations were made using the R 3.4.3 software and packages: nlme and car.

## Results

### Global Effect of the Intervention

The comparison between the experimental condition (i.e., EI training groups) and the control group showed that the intervention had significant positive effects on emotional competences. Particularly, the LME models showed that the EI training program helped the players belonging with the experimental condition to better express [*β* = +0.33, *t*(80) = 2.01, *p* = 0.048, ES = 0.28] and regulate their own emotions [*β* = +0.39, *t*(80) = 2.15, *p* = 0.035, ES = 0.21]. It could be also noted that the models showed a negative single effect of Time regarding the identification of others’ emotions [*β* = −0.16, *t*(82) = −2.61, *p* = 0.011, ES = 0.21]. No other significant or marginal effect has been found on the other emotional competences or on the global score.

### Specific Effect of the Intervention Considering the Characteristics of the “Trainer”

When EI training was implemented by the expert in sport psychology, LME models revealed a significant interaction positive effect of Condition by Time for regulation of own emotions [*β* = +0.07, *t*(40) = 2.21, *p* = 0.033, ES = 0.10]. A simple negative effect of Condition was also found for this competence (*p* = 0.008).

When EI training was implemented by the coach, LME models revealed a marginal positive interaction effect of condition by time for the expression of own emotions [*β* = +0.06, *t*(38) = 1.96, *p* = 0.055, ES = 0.09]. A simple negative effect of the Condition was also found for this competence (*p* = 0.015).

When EI training was implemented by the physiologist, LME models revealed a positive interaction effect of condition by time for the use of others’ emotions [*β* = 0.08, *t*(37), *p* = 0.033, ES = 0.15]. A simple negative effect of Condition was also found for this same competence (*p* = 0.002).

## Discussion

The aim of this study was to investigate the influence of EI trainer status on EI training effectiveness, regarding a short-term EI training program realized during a training camp before a competition. We first hypothesized that the current EI training would improve emotional competences in comparison to the control condition. This hypothesis was partially validated, with EI training specifically improving expressing and regulating one’s own emotions. Further, we hypothesized that, in comparison to a coach or physiologist, the EI training would trigger more improvements in emotional competences when the person delivering the intervention was an expert in sport psychology. Our findings did not support this hypothesis, as a positive effect was found on specific emotional competences for each experimental group.

Regarding our main hypothesis, only two (identification of own’s emotions and expressing one’s emotions) of the 10 emotional competences measured by the PEC (Brasseur et al., 2013) were shown to increase after the intervention. These results are in line with previous EI training research realized with rugby players (Campo et al., 2017), where improvements were shown only on some EI subscales (social competence, emotion perception, and emotion management). This can be explained by several factors, the main one being the duration of the EI intervention, which lasted less longer than interventions that have been shown to improve global EI (Hodzic et al., 2017; Kotsou et al., 2019). Longer interventions usually target the different emotional competences with more depth and potentially include follow-up (e.g., with emails) that help to integrate the elements learned. In addition, we have to acknowledge that measuring the effects of this intervention only with the PEC was finally non-optimal given the imposed time constraints of this protocol that limited evolutions at the trait level. Accordingly, these findings can already be seen as promising, given we were able to observe positive changes at the trait level within 1 week. Future studies should consider assessing the effectiveness of EI training not only at the trait level but also at the ability level, given this is what has primarily been trained during the current EI training based on the tripartite model (Mikolajczak, 2009).

With the benefit of the hindsight, a closer look to the context is required to better understand our findings. National teams are composed by players coming from different clubs. Except for the year of the World Rugby Cup, international seasons include only few matches played intermittently during the year. Most of the time, staff of national teams has 1 or 2 weeks only with international players to prepare a game. This implies great training structure constraints as coaches have to include different physical conditioning sessions and train the players to perform well in the different areas of their game plan. Moreover, while psychological resources are more and more often considered as a key factor of performance in rugby (Mellalieu et al., 2008; Campo et al., 2012; Campo and Djaït, 2016), mental training is not always shared among coaches as a trainable performance area and is still considered as being the “private preserve” of experts in sport psychology, implying consequently other additional organizational constraints. This observation has already been reported by Campo et al. (2015, 2016b) who explained that their individual EI training program was not adapted to the on-the-ground realities if it is expected to train all team members. The EI training program of Campo et al. (2017) was particularly time-consuming, which alters players’ compliance, increase the risk of players’ withdrawal and ultimately, the overall expected effects on performance. Therefore, the present study addressed this issue by testing the effects of an EI training program adapted to the schedule constraints imposed by the head coach of a national rugby union team. In addition, we sought to know whether a trained member of the staff may help players to increase their emotional competences. The current research demonstrated that it is possible to develop players’ emotional competences during a preparation competitive week without altering the technical and physical training habits.

Of course, these results should be also considered in the light of different other variables that may have influenced the observed evolutions. For instance, it is important to keep in mind that sport performance is a holistic process involving transactional relationships between physiological and psychological parameters. Thus, while some studies showed an influence of EI on physiological variables (Laborde et al., 2011, 2014, 2017b, 2018b), it should be also noted that other research have provided evidence about the effect of genetic substrates in the management of the sport stress situations (e.g., Petito et al., 2016). In addition, the weak experience at the elite level of our population raises the question of the adaptive changes in young athletes. Indeed, different studies showed differences in abilities to cope with stressful situation according to the experience and age (e.g., Brummer et al., 2013). Therefore, we invite researchers to have these perspectives in mind for future research testing the effectiveness of EI training programs.

Moreover, our findings invite to question the influence of the important constraints the head coach imposed for this study specifically. Indeed, learning processes require a familiarization time with the training program and a period of time to acquire the knowledge and facilitate the use of the tools taught. Because of the scheduling constraints (i.e., one 1-h session per day during the last 3 days of the week before the game), the players had not enough time to put into practice what they learn during the IE training sessions. Moreover, the program comprised two sessions on intrapersonal emotional competencies, while only one was devoted to improve players’ interpersonal emotional competencies. Therefore, future studies may consider designing longer EI trainings including one more session on interpersonal emotional competencies and lasting one or two “rest-days” between sessions, giving players the possibility to put their emotional competences into practice (intersession work). Increasing EI training program length and content should help to close the gap with EI training programs that have been shown to be effective in the literature (Hodzic et al., 2017; Kotsou et al., 2019).

Despite these limitations, the current study had several strengths. This is the first in sports to investigate the effects of the trainer delivering the EI training program. Indeed, while it is commonly accepted that such psychological intervention should be implemented by an expert in sport psychology, there is a lack of knowledge in the current EI literature on whether a person trained to deliver an EI training protocol may be also effective in improving players’ emotional competences. This echoes the quality of the relationship between practitioners and players. In the sport setting, it is largely accepted that the coach has a specific proximal relationship with the players. Previous research showed that the coach has a significant influence on athletes’ psychological states (e.g., to such extent that some academics metaphorically explained this influence by claiming that “the coach is the “first” mental trainer of the players and the team,” Campo and Djaït, 2016, p. 3). So, it could be expected that the coach, by using the same language as the players, and with his/her status may strengthen the players’ involvement in the training program. The same could be expected from the physiotherapist, who is also often involved in providing social support to the players. In the current study, the expert in sport psychology was able to improve players’ emotional regulation competences; the coach improved the players’ capacities to express their emotions, while the physiologist improved their competences to use others’ emotions. Therefore, our findings showed for the first time that the effects of the EI training were influenced by the person who delivered the program. This result presents a particular interest in highlighting the importance of the concept of working alliance, established in the field of therapy and counseling research (Hersoug et al., 2001; McKenna and Davis, 2009) and consisting in a close relationship between coach and coachee, here, athletes. The importance of proximity between coach and athletes has already been highlighted by different academics in the field of performance (e.g., Jowett and Poczwardowski, 2007) and should now be furthered in that of IE in sports.

From an applied perspective, our findings open new venues in implementing mental training in sport, by highlighting the suitability of a group-based approach in the training-week structure and demonstrating positive effects in a short-time period. Moreover, the fact that a coach previously trained to deliver EI training may be able to develop emotional competences in his/her players is encouraging, given a staff member may be more autonomous in providing this kind training in a constantly changing schedule. While mobilizing at the same time almost 100 elite players in four homogenous experimental groups may be considered as a strength of an IE intervention study in the context of sport (Laborde et al., 2016), we also acknowledge that the few number of participants is also a limitation to well support and generalize the findings. Therefore, we invite future researchers to test with a wider sample these preliminary results. By the meantime, they provide first evidence suggesting the integration of EI trainings in the preparation periods during international seasons.

## Data Availability

The datasets generated for this study are available on request to the corresponding author.

## Ethics Statement

In the university of MC in which the experiment was carried out, no ethical board existed for non-invasive studies. Nonetheless, to ensure a high ethical standard for our research, we presented the study to a panel of independent researchers in the field of the humanities. This consortium informed us that this study respected the international ethical guideline of the World Medical Association Declaration of Helsinki (2013). This has been noted as follows in the manuscript: “Following approval by a consortium of independent researchers in the humanities and in sport sciences verifying that the study procedures followed international ethical guidelines for research (World Medical Association Declaration of Helsinki, 2013), […]. Players who agreed to participate in the study provided informed consent.”

## Author Contributions

MC led the project, analyzed the data, and wrote this manuscript. SL participated actively in the writing of the manuscript and contributed in the elaboration of the protocol. GM participated in the data analysis strategy, the elaboration of the protocol and contributed in the manuscript. BL and MN contributed equally in the manuscript and in the elaboration of the protocol.

### Conflict of Interest Statement

The authors declare that the research was conducted in the absence of any commercial or financial relationships that could be construed as a potential conflict of interest.
